# Increased platelet expression of FcGammaRIIa and its potential impact on platelet reactivity in patients with end stage renal disease

**DOI:** 10.1186/1477-9560-5-7

**Published:** 2007-06-04

**Authors:** Feliciano A Serrano, Mohamed El-Shahawy, Richard J Solomon, Burton E Sobel, David J Schneider

**Affiliations:** 1 Department of Medicine, University of Vermont, Burlington, Vermont, USA; 2 Department of Medicine, University of Southern California, Los Angeles, California, USA

## Abstract

**Background:**

Increased platelet reactivity has been implicated in cardiovascular disease – the major cause of death in patients with end stage renal disease (ESRD). FcGammaRIIA is a component of glycoprotein VI and Ib-IX-V that mediate activation of platelets by collagen and von Willebrand factor. To determine whether expression of FcGammaRIIA impacts platelet reactivity we quantified its expression and platelet reactivity in 33 patients with ESRD who were undergoing hemodialysis.

**Methods:**

Blood samples were obtained from patients immediately before hemodialysis and before administration of heparin. Platelet expression of FcGammaRIIA and the activation of platelets in response to low concentrations of convulxin (1 ng/ml, selected to mimic effects of collagen), thrombin (1 nM), adenosine diphosphate (ADP, 0.2 uM), or platelet activating factor (PAF, 1 nM) were determined with the use of flow cytometry in samples of whole blood anticoagulated with corn trypsin inhibitor (a specific inhibitor of Factor XIIa).

**Results:**

Patients were stratified with respect to the median expression of FcGammaRIIA. Patients with high platelet expression of FcGammaRIIA exhibited 3-fold greater platelet reactivity compared with that in those with low expression in response to convulxin (p < 0.01) and 2-fold greater activation in response to thrombin, ADP, and PAF (p < 0.05 for each). For each agonist, expression of FcGammaRIIA correlated modestly but positively with platelet reactivity. The strongest correlation was with thrombin-induced activation (r = 0.6, p < 0.001).

**Conclusion:**

Increased platelet reactivity in response to low concentrations of diverse agonists is associated with high expression of FcGammaRIIA and may contribute to an increased risk of thrombosis in patients with ESRD.

## Background

Cardiovascular disease is the major cause of death in patients with end stage renal disease (ESRD) accounting for nearly 50% of deaths [1]. Patients undergoing long-term dialysis have a particularly poor rate of survival after myocardial infarction [2]. We and others have found that platelet reactivity (i.e. the propensity of platelets to activate) is increased in patients with ESRD undergoing hemodialysis [3-5]. Increased platelet reactivity has been associated with an increased risk of subsequent cardiac events [6-8]. Accordingly, one factor contributing to a greater risk of cardiovascular disease and death in patients with ESRD may be increased platelet reactivity.

Numerous Fc receptors are known. All are members of the immunoglobulin superfamily. Human platelets express one, an FcγR encoded by the FcγRIIa gene [9]. FcγRIIa is a component of both the glycoprotein (GP) VI receptor that mediates activation of platelets by collagen [10] and the GP Ib-IX-V receptor that mediates activation of platelets by von Willebrand Factor [11]. Increased platelet expression of FcγRIIa has been seen in patients with diabetes [12] and ESRD [13], conditions known to be at an increased risk for cardiovascular disease. Increased expression has been observed in patients who have experienced coronary or cerebral thrombosis [14] and was associated with a greater incidence of arterial thrombotic events in patients with ESRD [13]. FcγRIIa appears to participate in thrombotic complications associated with the severe form of heparin-induced thrombocytopenia/thrombosis (HITT) [15].

The present study was performed to determine whether platelet expression of FcγRIIa correlates with platelet reactivity in patients with ESRD undergoing hemodialysis. Expression of FcγRIIa and platelet reactivity were determined with the use of flow cytometry. Platelet reactivity was determined in response to convulxin (a snake venom that mimics the effects of collagen on GP VI [16]) as well as to adenosine diphosphate (ADP), thrombin, or platelet activating factor (PAF).

## Methods

### Subjects

In a protocol approved by the University of Vermont Institutional Review Board, 33 subjects were enrolled after written informed consent had been obtained. Eligible patients were those of more than 18 years of age who were undergoing hemodialysis for ESRD. Patients were excluded if they had an intercurrent illness such as pneumonia or congestive heart failure, any hematological disorder, a terminal illness with expected survival of less than 6 months, or the inability to provide informed consent. No patient had experienced a recent (within 1 month) thrombotic event.

### Collection of blood samples

Blood samples were obtained from the dialysis catheter immediately after its insertion into the arterial portion of the arteriovenous fistula and before administration of an anticoagulant. We have found that taking blood from a catheter does not per se influence assessment of platelet function [3,6]. All blood samples were drawn into syringes containing corn trypsin inhibitor (CTI, 32 μg/ml, 1:10 V/V, Enzyme Research, South Bend, IN) with the use of the two-syringe technique. CTI is a specific inhibitor of coagulation factor XIIa [17] and was used as the anticoagulant to avoid altered activation of platelets by conventionally used anticoagulants such as citrate [18].

### Determination of platelet reactivity

Assays were performed by adding 5 μl of whole blood to microcentrifuge tubes containing 60 μl of HEPES-Tyrodes buffer (5 mM HEPES, 137 mM NaCl, 2.7 mM NaHCO_3_, 0.36 mM NaH_2_PO_4_, 2 mM CaCl_2_, 4 mM MgCl_2_, and 5 mM dextrose, pH 7.4), fluorochrome-labeled ligands, the peptide GPRP (Gly-Pro-Arg-Pro) to prevent polymerization of endogenous fibrinogen [19] and agonist. A peridinin chlorophyll protein (per-CP) conjugated antibody to GP IIIa (CD61, Becton Dickinson, San Jose, California) was used as an activation-independent marker of platelets. This antibody does not inhibit binding of fibrinogen to the activated conformer of GP IIb-IIIa. A fluorescein isothiocyanate (FITC) conjugated PAC-1 antibody (Becton Dickinson) was used to assess activation (conformation change) of GP IIb-IIIa. A phycoerythrin (PE) conjugated antibody to P-selectin (CD62, Becton Dickinson) was used to assess surface expression of P-selectin associated with α-granule degranulation. We used concentrations of each agonist that induce sub-maximal activation because we have shown previously that such concentrations effectively delineate inter-individual differences in platelet reactivity [6]. Agonists included thrombin (1 nM, Haematologic Technologies Inc, Essex Junction, VT), ADP (0.2 μM, BioData, Horsham PA), PAF (1 nM, Sigma, St. Louis, MO), and convulxin (1 ng/ml, Pentapharm, Basel Switzerland)

The reaction mixture was incubated at room temperature for 15 minutes. Subsequently, platelets were fixed, and the red cells were lysed by the addition of 100 μl of Optilyse-C (1.5% formadehyde, Immunotech, Wesbrook, Maine). All assays were performed in duplicate. We have demonstrated previously that the intra-assay coefficient of variation is less than 10% [20]. To assess the extent of nonspecific association of proteins with platelets, blood was added to control tubes with FITC-labeled and PE-labeled non-immune immunoglobulins. Flow cytometric analysis was performed with a fluorescence-activated cell sorter (Epics Elite EPS, Coulter). The population of platelets was identified on the basis of particle size (forward and 90° side scatter) and the association with CD 61 antibody. The control ligands (non-immune immunoglobulins) were used for determination of a threshold above which activation-dependent binding was present. Platelets for which binding exceeded the threshold were identified as activated, and results were reported as the percentage of platelets activated with respect to binding of PAC-1 and surface expression of P-selectin.

### Expression of FcγRIIa

Platelet expression of FcγRIIa was quantified with the use of flow cytometry. Blood (5 μl) was added to 60 μl of HEPES-Tyrodes buffer (described above) plus antibodies. Platelets were identified as described above on the basis of size and with the use of a PerCP-conjugated anti-CD61. FcγRIIa expression was quantified with a primary goat anti-Fcγ RIIA/CD32a (0.2 mg/ml, R&D Systems, Minneapolis, MN) and a secondary PE-conjugated anti-goat IgG (10:1 ratio of secondary to primary antibody, Jackson ImmunoResearch Laboratories, West Grove PA). Assay of the expression of FcγRIIa was performed in duplicate. A control assay tube that contained only secondary antibody was used to quantify non-specific association of secondary antibody with platelets. Mean fluorescence intensity (MFI) was used to quantify expression of FcγRIIa, and the expression of FcγRIIa was quantified by subtracting the MFI in the control tube from the average total MFI determined in the assay tubes containing both primary and secondary antibodies. Preliminary experiments were performed to demonstrate that the concentration of primary antibody and the ratio of primary to secondary antibody yielded the greatest sensitivity for detection of expression of FcγRIIa.

### Statistical analysis

Values are means ± SD. Differences in platelet function were identified with the use of a Student's t tests. Categorical variables were compared with the use of chi squared analysis. Significance of linear regressions was assessed with the use of analysis of variance. Significance was defined as p < 0.05.

## Results

**Figure 1:**
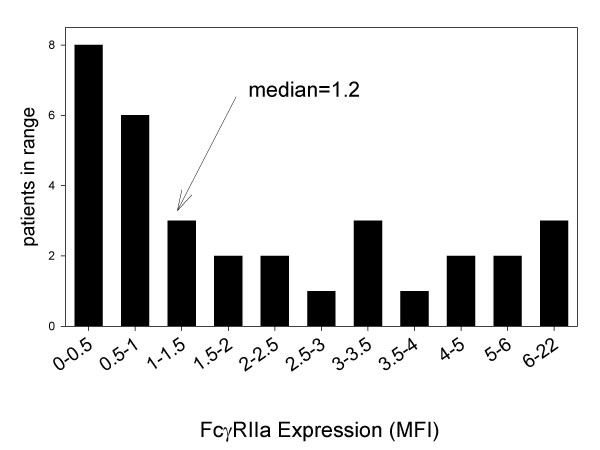
Expression of FcγRIIa by platelets from 33 patients with ESRD undergoing hemodialysis. Expression of FcγRIIa was determined with the use of flow cytometry. FcγRIIa MFI was quantified by subtracting the mean fluorescence intensity (MFI) seen with secondary antibody alone from the MFI seen with both the primary and secondary antibody. A skewed distribution is apparent with 50% of the patients exhibiting low expression (MFI<1.2).

The clinical characteristics of the patients studied are shown in table 1. Expression of FcγRIIa by platelets from the patients studied is shown in figure 1. A skewed distribution of expression of FcγRIIa is evident with 50% of the patients exhibiting an MFI less than 1.2. By contrast, platelet expression of FcγRIIa ranged from 1.2 to 22 in the remaining patients.

**Table 1:** Clinical characteristics of patients

Characteristic	Low FcγRIIa	High FcγRIIa	
	n (%)	n (%)	p value
Age (mean ± SD)	67 ± 15	67 ± 12	1
male	8 (50%)	10 (63%)	0.87
CAD	11 (71%)	10 (63%)	0.82
CVA/TIA	2 (12%)	7 (44%)	0.15
Hypertension	13 (76%)	16 (100%)	0.55
Diabetes	10 (61%)	10 (63%)	0.89
Hyperlipidemia	11 (65%)	9 (56%)	0.57
Treatment with			
aspirin	9 (53%)	11(69%)	0.89
warfarin	3 (18%)	3 (19%)	0.71
thienopyridine	3 (18%)	5 (31%)	0.64
β blocker	8 (47%)	11 (69%)	0.36
Calcium blocker	7 (41%)	5 (31%)	0.81
ACEI/ARB	7 (41%)	4 (25%)	0.55
insulin	5 (29%)	7 (44%)	0.59
oral hypoglycemic	3 (18%)	3 (19%)	0.71
Duration of HD (mo – mean ± SD)	31 ± 25	32 ± 23	0.91

CAD = coronary arterial disease, CVA = cerebrovascular accident, TIA = transient ischemic attack, ACEI = angiotensin converting enzyme inhibitor, ARB = angiotensin receptor blocker, HD = hemodialysis, mo = month

### Platelet reactivity

**Figure 2:**
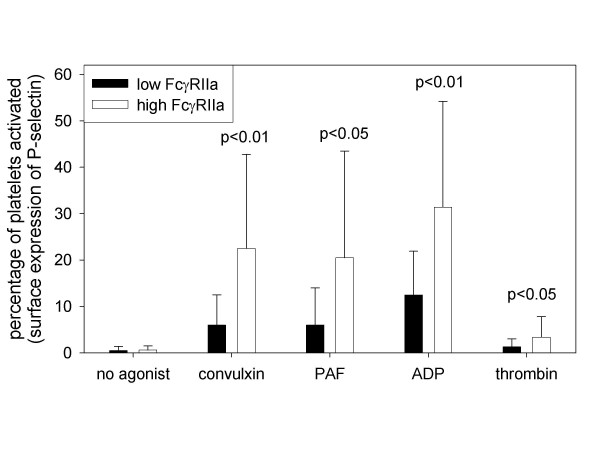
Activation of platelets identified by surface expression of P-selectin. Patients with ESRD (n = 33) were stratified into 2 groups on the basis of median expression of FcγRIIa. Activation of platelets was quantified with the use of flow cytometry for delineation of the percentage of platelets expressing P-selectin on their surface in the absence of agonist and in response to the collagen mimetic convulxin (1 ng/ml), PAF (1 nM), ADP (0.2 μM), or thrombin (1 nM). Patients with high expression of FcγRIIa did not exhibit greater activation of platelets in the absence of agonist but did exhibit greater activation in response to each of the agonists used.

**Figure 3:**
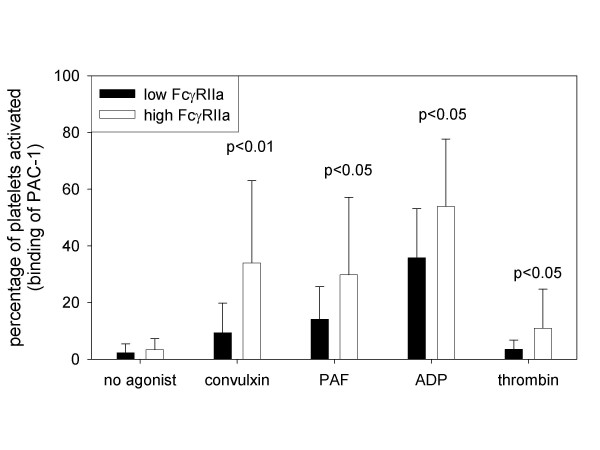
Activation of platelets identified by the binding of the antibody PAC-1 to the activated conformer of GP IIb-IIIa. Patients with ESRD (n = 33) were stratified into 2 groups on the basis of median expression of FcγRIIa. Activation of platelets was quantified with the use of flow cytometry to define the percentage of platelets that bound PAC-1 in the absence of agonist and in response to the collagen mimetic convulxin (1 ng/ml), PAF (1 nM), ADP (0.2 μM), or thrombin (1 nM). Patients with high expression of FcγRIIa did not exhibit greater activation of platelets in the absence of agonist but did exhibit greater activation in response to each of the agonists used.

Patients were stratified into 2 groups on the basis of the median expression of FcγRIIa. Activation of platelets was identified by both the surface expression of P-selectin (figure 2) and the binding of PAC-1 to the activated conformer of GP IIb-IIIa (figure 3). Activation of platelets in response to the collagen mimetic, convulxin, was 3-fold greater in the patients with high FcγRIIa expression (p < 0.01, figure 2 and 3). Increased expression of FcγRIIa was associated with approximately 2-fold greater platelet reactivity in response to ADP, thrombin, or PAF (p < 0.05 for each, figure 2 and 3). Accordingly, patients with high FcγRIIa expression exhibited greater platelet reactivity in response to each of the agonists.

**Figure 4:**
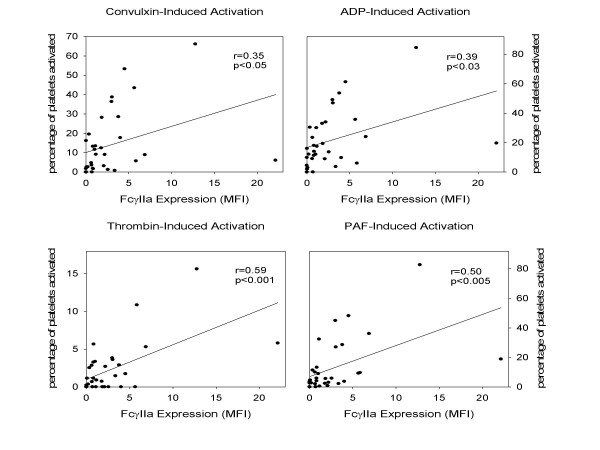
The correlation of platelet reactivity with expression of FcγRIIa. Activation of platelets was identified with the use of flow cytometry to delineate surface expression of P-selectin in response to exposure of the platelets to the collagen mimetic convulxin (1 ng/ml), PAF (1 nM), ADP (0.2 μM), or thrombin (1 nM). Expression of FcγRIIa was quantified with the use of flow cytometry by subtracting the MFI seen with secondary antibody alone from the MFI seen with both the primary and secondary antibody. Significant and similar, although modest correlations (r values ranging from approximately 0.4–0.6) were seen with each of the agonists used.

Diverse genetic and environmental stimuli influence platelet reactivity. Nevertheless, platelet reactivity in response to each of the agonists correlated modestly but significantly with platelet expression of FcγRIIa (figure 4). The strongest correlation was with thrombin-induced activation (r = 0.6, p < 0.001).

## Discussion

Our results demonstrate that platelet expression of FcγRIIa varies substantially in patients with ESRD undergoing hemodialysis. Distribution of platelet expression of FcγRIIa was skewed with some patients exhibiting substantially greater platelet expression of FcγRIIa. Greater expression of FcγRIIa was associated with an increased propensity for activation (i.e. increased platelet reactivity) in response to each agonist tested. These results suggest that in addition to its direct participation in the activation of platelets induced by collagen and von Willebrand factor, greater FcγRIIa expression increases platelet reactivity. Increased platelet reactivity has been associated with a greater risk of cardiovascular events [6-8,13]. Accordingly, our results demonstrating an association between greater platelet expression of FcγRIIa and increased platelet reactivity are consistent with a previous report demonstrating that increased expression of FcγRIIa is associated with a greater risk of arterial thrombotic events in patients with ESRD [13].

FcγRIIa is a component of both the GP VI and GP Ib-IX-V receptors on the platelet surface. GP VI is composed of two GP VI molecules and an FcγRIIa dimer [10]. GP VI forms a dimeric complex that exhibits high affinity binding to collagen. Activation of GP VI is initiated by tyrosine phosphorylation of the immunoreceptor tyrosine-based activation motif (ITAM) of FcγRIIa, and subsequent tyrosine phosphorylation of signaling proteins leads to activation of platelets. Activation of platelets by GP Ib-IX-V is not completely understood. FcγRIIa co-localizes with GP Ib-IX-V [21] and participates in but may not be essential to GP Ib-IX-V-induced activation of platelets [11]. Bacteria such as staphylococcus aureus and group B streptococcus can induce activation of platelets through direct interaction with FcγRIIa [22,23]. Thus, FcγRIIa can participate in the activation of platelets directly as well as by serving as a component of the receptors for collagen and von Willebrand factor.

Our results demonstrate that increased expression of FcγRIIa is associated with greater platelet reactivity in response to exposure of platelets to diverse stimuli including ADP, thrombin and PAF. We used convulxin to mimic the effects of collagen because collagen-induced activation is not efficient in our system which does not employ sheer forces or stirring [18]. Convulxin, like collagen, activates platelets through interaction with GP VI [16]. Consistent with the pivotal role of FcγRIIa in GP VI mediated activation of platelets as well as the results obtained by others with collagen [12,14], we saw greater activation of platelets by convulxin when the expression of FcγRIIa was increased. Greater activation in response to ADP, thrombin, and PAF was unexpected. The similar, albeit modest, correlation between platelet expression of FcγRIIa and activation in response to each of the agonists suggests that the effects of FcγRIIa on the activation of platelets are not confined to those mediated by interactions involving the GP VI or the GP Ib-IX-V receptor but rather influence activation by diverse receptors. Evidence that bacteria can directly activate platelets through direction interaction with FcγRIIa [22,23] and that FcγRIIa may not be essential to GP Ib-IX-V-induced activation of platelets [11], demonstrate that expression of FcγRIIa is not solely a reflection of the density of GP VI and GP Ib-IX-V. These observations in combination with our results suggest that platelet expression of FcγRIIa has an independent influence on platelet reactivity.

FcγRIIa has not been reported to be a component of signaling induced by ADP receptors (P2Y1 or P2Y12), thrombin receptors (protease activated receptor [PAR] 1 and 4) or the PAF receptor. Nevertheless, the phenomenon of increased activation induced by multiple agonists has been recognized previously. For example, maximal activation of the GTPase Rac [24] and maximal activation of platelets occur when both FcγRIIa and the P2Y12 receptor are activated [25]. These observations suggest that ADP released by platelets [26] when they are activated may contribute to activation induced by agonists that activate FcγRIIa and are consistent with the contribution of ADP to thrombin-induced activation of platelets [27].

We did not see greater activation of platelets in the absence of agonist in patients with increased expression of FcγRIIa. A limited (< 5%) and similar number of platelets in patients with high and patients with low expression of FcγRIIa exhibited evidence of activation in the absence of agonist. Our results are consistent with those in a recent report demonstrating that FcγRIIa participates in the activation of platelets induced by low but not high concentrations of thrombin [28]. Thus, it appears that increased expression of FcγRIIa primes platelets for activation by low concentrations of diverse agonists.

Our results were obtained with blood from 33 subjects. The limited sample size prevents definitive exclusion of potentially confounding variables. Additional studies with more patients and correlation with the incidence of clinical events will be necessary to determine the impact of platelet expression of FcγRIIa on platelet function and the risk of arterial thrombotic events.

The mechanism(s) responsible for high expression of FcγRIIa that was seen in 50% of our patients with ESRD was not determined. Although genetic predisposition may contribute, the high expression seen in patients with ESRD [13] as well as those with acute coronary and cerebrovascular events [14] and those with diabetes [12] implicates factors other than genetics as determinants of expression. Neutrophil expression of FcγRIIa is increased by tumor necrosis factor alpha [29]. Thus, expression of FcγRIIa may be regulated dynamically by multiple factors. Accordingly, elucidation of factors that increase expression of FcγRIIa in patients with ESRD treated with hemodialysis may identify novel pharmacologic targets that can decrease platelet reactivity and reduce the incidence of cardiovascular events.

## Conclusion

We found that patients with ESRD treated with hemodialysis who exhibit high platelet expression of FcγRIIa exhibit increased platelet reactivity. Increased platelet reactivity is associated with an increased risk of cardiac events [6-8,13]. Thus, our results are consistent with a previous report that found that increased platelet expression of FcγRIIa was associated with a greater incidence of arterial thrombotic events in patients with ESRD [13]. Pharmacologic modification of expression of FcγRIIa may be a promising prophylactic target.

## Competing interests

The author(s) declare that they have no competing interests.

## Authors' contributions

• FS participated in the design of the study, recruited patients, acquired the primary data, participated in the analysis of data and preparation of the manuscript

• ME-S participated in the conceptualization of the study as well as the analysis of data and preparation of the manuscript

• RS participated in the conceptualization of the study as well as the analysis of data and preparation of the manuscript

• BS participated in the design of the study as well as the analysis of data and preparation of the manuscript

• DS participated in the conceptualization and design of the study as well as the analysis of data and preparation of the manuscript

All authors read and approved the final manuscript
